# Vocal-cord Only vs. Complete Laryngeal radiation (VOCAL): a randomized multicentric Bayesian phase II trial

**DOI:** 10.1186/s12885-021-08195-8

**Published:** 2021-04-22

**Authors:** Houda Bahig, David I. Rosenthal, Félix-Phuc Nguyen-Tan, David C. Fuller, Ying Yuan, Katherine A. Hutcheson, Apostolos Christopoulos, Anthony C. Nichols, Kevin Fung, Olivier Ballivy, Edith Filion, Sweet Ping Ng, Louise Lambert, Jennifer Dorth, Kenneth S. Hu, David Palma

**Affiliations:** 1 Radiation Oncology Department, Centre Hospitalier de l’Université de Montréal, 1051 Sanguinet, Montreal, QC H2X 3E4 Canada; 2 Radiation Oncology Department, University of Texas MD Anderson Cancer Center, 1515 Holcombe, Houston, TX 77030 USA; 3 Biostatistics Department, University of Texas MD Anderson Cancer Center, Houston, USA; 4 Head and Neck Surgery Department, University of Texas MD Anderson Cancer Center, Houston, USA; 5 Head and Neck Surgery Department, Centre Hospitalier de l’Université de Montréal, Montreal, Canada; 6 Department of Otolaryngology - Head and Neck Surgery, Western University, London, Ontario Canada; 7 Radiation Oncology Department, Peter MacCallum Cancer Centre, Melbourne, Australia; 8 Radiation Oncology Department, Centre Intégré de Cancérologie de Laval, Laval, Canada; 9 Radiation Oncology Department, Case Western Reserve University, Cleveland, USA; 10 Radiation Oncology Department, NYU Langone Health, Newyork, USA; 11 Radiation Oncology Department, Western University, London, Ontario Canada

**Keywords:** Glottic cancer, Larynx, Radiotherapy, Vocal cord, Local control

## Abstract

**Background:**

Radiotherapy, along with laser surgery, is considered a standard treatment option for patients with early glottic squamous cell cancer (SCC). Historically, patients have received complete larynx radiotherapy (CL-RT) due to fear of swallowing and respiratory laryngeal motion and this remains the standard approach in many academic institutions. Local control (LC) rates with CL-RT have been excellent, however this treatment can carry significant toxicities include adverse voice and swallowing outcomes, along with increased long-term risk of cerebrovascular morbidity. A recent retrospective study reported improved voice quality and similar local control outcomes with focused vocal cord radiotherapy (VC-RT) compared to CL-RT. There is currently no prospective evidence on the safety of VC-RT. The primary objective of this Bayesian Phase II trial is to compare the LC of VC-RT to that of CL-RT in patients with T1N0 glottic SCC.

**Methods:**

One hundred and fifty-five patients with T1a-b N0 SCC of the true vocal cords that are n ot candidate or declined laser surgery, will be randomized in a 1:3 ratio the control arm (CL-RT) and the experimental arm (VC-RT). Randomisation will be stratified by tumor stage (T1a/T1b) and by site (each site will be allowed to select one preferred radiation dose regimen, to be used in both arms). CL-RT volumes will correspond to the conventional RT volumes, with the planning target volume extending from the top of thyroid cartilage lamina superiorly to the bottom of the cricoid inferiorly. VC-RT volumes will include the involved vocal cord(s) and a margin accounting for respiration and set-up uncertainty. The primary endpoint will be LC at 2-years, while secondary endpoints will include patient-reported outcomes (voice impairment, dysphagia and symptom burden), acute and late toxicity radiation-induced toxicity, overall survival, progression free survival, as well as an optional component of acoustic and objective measures of voice analysis using the Consensus Auditory-Perceptual Evaluation of Voice.

**Discussion:**

This study would constitute the first prospective evidence on the efficacy and safety of VC-RT in early glottic cancer. If positive, this study would result in the adoption of VC-RT as standard approach in early glottic cancer.

**Trial registration:**

ClinicalTrials.gov Identifier: NCT03759431

Registration date: November 30, 2018

**Supplementary Information:**

The online version contains supplementary material available at 10.1186/s12885-021-08195-8.

## Background

### Role of radiotherapy in early glottic cancer

Each year, over 150,000 new cases of laryngeal cancer are diagnosed worldwide [1] and over a thousand new cases are diagnosed in Canada [2]. Treatment options for early stage laryngeal cancer (T1–2N0) include transoral endoscopic microsurgery [3–5], radical radiotherapy (RT) [6–9] and, in rare cases, partial laryngectomy via open surgery [10–12]. Whereas endoscopic microsurgery (with or without laser) typically involves resection of only the tumor-bearing vocal cord with a narrow 1–2 mm margin, current standard RT involves irradiation of the entire larynx [13–15]. Although prospective evidence comparing RT to surgery is lacking, both treatment options were reported to have equivalent oncological outcomes in terms of local control (LC) and overall survival (OS) in several meta-analysis [16–18] and systematic reviews [19–23]. Radical RT outcomes for early glottic cancer are excellent with reported 5-year LC rates varying between 85 and 95% and 5-year OS exceeding 90% [6–8, 24, 25]. Although it is often suggested that RT is associated with better voice preservation compared to surgery, notably in larger tumors and tumors involving the anterior commissure [26], this remains controversial [19]. In a first meta-analysis including 8 retrospective cohort studies, although 2 studies reported improved voice preservation with RT, pooled results showed similar voice outcomes between transoral laser surgery and RT in T1 glottic cancers [27]. In a more recent meta-analysis including 14 studies and comparing outcomes of surgery vs. RT for T1 glottis cancers, while subjective voice assessments were comparable, RT was associated with improved maximum phonation time and decreased fundamental frequency [28]. Selection of optimal treatment modality currently involves institutional expertise and elicitation of patient preferences, and although substantiated by debatable evidence, also takes into account factors such as tumor size, location and histology [29–31]. In a recent prospective study on the choice of treatment in patients with T1–2N0 glottic cancer, it was reported while 51% of patients are oriented directly towards RT by the medical team (i.e. deemed not suitable for surgery), a third of patients offered either transoral laser surgery or RT opted for RT [32].

### Toxicities of complete larynx radiotherapy

Despite good LC, a substantial number of patients treated with radical RT experience persistent voice impairment after treatment [33]. Several studies have reported mild-to-moderate voice impairments after early glottic cancer RT in the acute, subacute and chronic settings [27, 34–36]. There is currently limited data on threshold doses for functional voice preservation and, in the context of current RT fields, this has been of little pertinence considering that the entire larynx would receive the full dose, leaving little room for organs at risk sparing. Dornfeld et al. [37] found a strong correlation between quality of speech and doses to various structures of the glottic and supraglottic larynx as well as the pharyngeal walls. Available dose-volume data suggest that mean dose to the larynx above 45 Gy and mean dose to the non-involved vocal cord above 50 Gy are predictors of grade ≥ 2 laryngeal oedema and worse voice outcomes [38, 39].

Other common toxicities of larynx RT for early glottic cancer include increased risk of carotid artery stenosis and hypothyroidism. Cerebrovascular morbidity from carotid artery stenosis has been documented in several studies in the setting of conventional RT [40–42]. In a recent SEER-database study, RT for treatment of early glottic cancer was associated with an increased risk of mortality due to cerebrovascular events in comparison to surgery [42]. Doses to the carotids between 35 and 50 Gy have been associated with carotid vessels wall thickening [43]. In addition, rates of radiation-induced hypothyroidism vary between 13 and 47% [35, 44], with highest frequency at 1 year after treatment. Other severe toxicities of larynx RT include less than 1% risk of permanent tracheostomy due persistent laryngeal oedema and loss of functional larynx [35, 45] and less than 1% risk of persistent mild or moderate dysphagia [46]. Importantly, laryngeal cancer patients have 22% risk of developing a secondary malignancy, with the wide majority originating from the upper aero-digestive tract [47]. In this context, their previous history of complete larynx RT limits their future therapeutic options.

### Complete larynx radiation field and larynx motion

The historical standard for early glottic cancer remains the use of 3D-conformal radiotherapy, most commonly using lateral opposing fields, with field size of 5 × 5 cm^2^ to 6 × 6 cm^2^ [13, 14] centered on the thyroid cartilage. Typically, the superior, posterior, inferior and anterior borders of the field are 0.5–1.0 cm above the thyroid notch, 1 cm behind the thyroid cartilage, below the cricoid and 1 cm beyond the patient’s external contour, respectively [13]. Although the use of intensity modulated radiotherapy (IMRT) for early glottic cancer remains controversial, several institutions have adopted carotid-sparing IMRT planning [15, 48, 49]. Clinical outcomes from complete larynx IMRT was published by the Memorial Sloan-Kettering Cancer Center and showed excellent 3 years LC [15]. The margins used in complete larynx IMRT remain conservative because of fear of geographical miss associated with internal motion of the larynx such as swallowing or breathing.

Although swallowing motion is associated with large larynx excursion up to 2 cm in the superior direction [50–53], swallowing motion was reported to be rare, rapid and easily suppressed by patients, and is therefore considered to have negligible impact on RT dose delivery [50, 53–56]. Although precaution should be taken to ensure that the planning CT is not acquired while the patient is swallowing (to avoid a risk of systematic error throughout treatment), additional margins to account for swallowing motion appear unnecessary [50]. On the other hand, respiratory motion in the order of several millimetres has also been described [54, 55]. Respiratory motion reaching 6 mm in the superior-inferior direction and 2 mm in the antero-posterior direction has previously been described [50]. Such intra-fraction motion cannot be addressed by means of daily image guidance and can be associated with a risk of tumor miss in the context of tighter treatment margin. In addition, occurrence of a larynx shift in relation to the vertebral structures, potentially resulting from anatomical changes over time or set-up reproducibility, has also been described [50, 57]. The latter finding stresses the importance of daily imaging with laryngeal match rather than bone match if tight margins are considered.

### Vocal-cord only irradiation

In recent years, increasing interest in reducing RT treatment volumes has emerged, with the objective of reducing toxicity while maintaining LC. In fact, in the era of IMRT and image-guided radiotherapy (IGRT), it is appealing to mirror surgical approaches and evaluate vocal-cord radiotherapy (VC-RT). Focal vocal cord RT for T1N0 glottic cancer has been assessed in dosimetric studies which confirmed adequate target volume coverage [58], homogeneity of planning target volume (PTV) coverage [50], as well as sparing of various laryngeal structures (including the contralateral vocal cord and arytenoid), the carotids and thyroid gland compared to conventional RT [50, 59, 60]. The group from Erasmus Medical Center Cancer has reported clinical outcomes from a retrospective analysis of 30 patients with T1aN0 glottic cancer treated with VC-RT [35]. They reported excellent 2 year LC of 100% as well as lower rates of acute toxicities and improved voice outcomes compared to a similar cohort of patients treated with CL-RT [35]. In fact, VC-RT was associated with clinically significant voice preservation compared to CL-RT immediately after RT, at 6–12 weeks and at 6–12-18 months after RT [36]. By maximally avoiding structures involved in voice preservation, VC-RT has the potential to reduce the rate and severity of acute and chronic toxicities and minimize voice impairment, while maintaining current excellent local control rates. While some institutions have adopted a partial larynx RT approach in early glottic cancer, there is currently no prospective evidence on efficacy or safety of VC-RT and many academic institutions continue to consider treating the entire larynx as standard of care. The hypothesis of the current phase II study is that VC-RT would lead to non-inferior LC compared to historical outcomes of CL-RT in patients with T1N0 glottic cancer.

## Methods/design

### Study objectives

The objective of this trial is to assess the efficacy and safety of VC-RT, compared to CL-RT, in T1N0 glottic squamous cell carcinoma.

Primary endpoint:

- Local control at 2-year after the end of RT.

*Local control will be defined as absence of biopsy proven recurrence within the larynx.*

Secondary endpoints:

- Patient-reported outcomes including dysphagia, voice impairment, and symptom burden.
- Acute and late toxicity radiation-induced toxicity
- OS and progression free survival (PFS)
- Acoustic and objective measures of voice analysis using the Consensus Auditory-Perceptual Evaluation of Voice.

### Study design

**Fig. 1:**
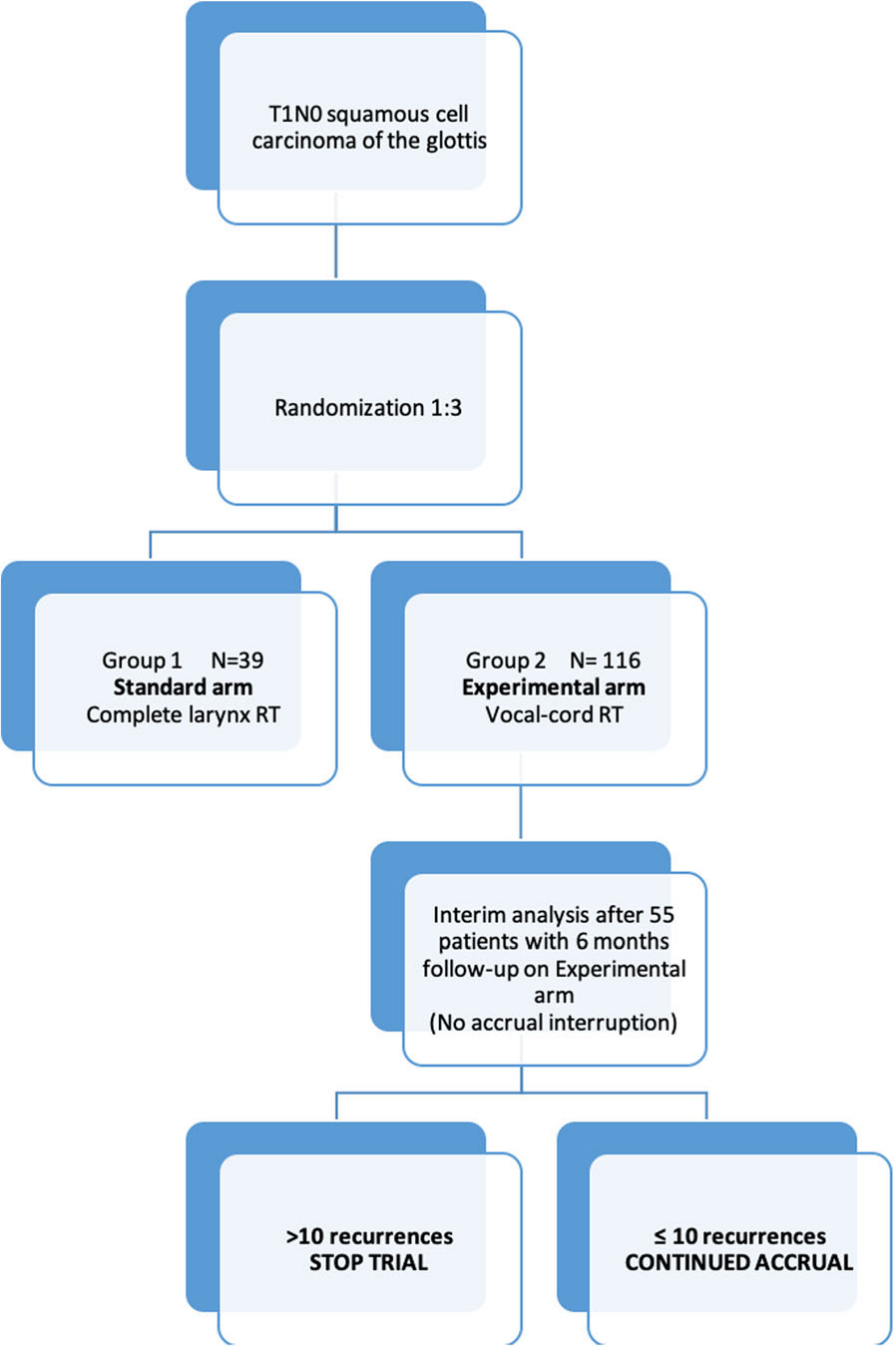
Study Scheme of the VOCAL Trial. N = Number of patients

We propose a phase II, multicentre Bayesian trial. A total of 155 patients will be randomized in a 1:3 ratio to CL-RT (39 patients) or VC-RT (116 patients). Patients will be stratified by tumor stage (T1a/T1b) and by site (Fig. 1). This study, which is registered on clinicaltrials.gov (NCT03759431), will include tertiary, academic hospitals in Canada and the United States. One dose and fractionation regimen will be determined by each participating center and will have to be the same in both arms. The study is powered to compare LC of VC-RT compared to historical outcomes of CL-RT. The purpose of the randomization will be to generate data on secondary outcomes of voice impairment, dysphagia and quality of life as well as survival outcomes in the control arm.

### Conditions for patient eligibility

- Age > 18 years
- Stage T1a-b N0 of the true vocal cords planned for definitive RT
- Patient not candidate for laser surgery or declined laser surgery
- Biopsy-confirmed squamous cell carcinoma, including verrucous carcinoma
- Eastern Cooperative Oncology Group (ECOG) performance status 0–2
- Ability to provide written informed consent.
- Ability to understand and read English or French at a level adequate for completion of patient reported outcomes questionnaires

### Conditions for patient ineligibility

- Previous irradiation of the head and neck region
- Pregnancy or breastfeeding
- Any medical condition that represents, in the opinion of the investigator, a contraindication to radiotherapy or would prevent follow-up after radiotherapy.
- Prior invasive malignancy (except non-melanomatous skin cancer) unless disease free for a minimum of 2 years.

### Required pre-treatment evaluation

- History and Physical Examination, including:
  - ° Laryngoscopy, with detailed diagram of the primary lesion confirmed by both the radiation oncologist and head and neck surgeon
  - ° Biopsy of the primary tumor
- Patient reported outcomes questionnaires including the Voice Handicap Index 10 (VHI-10), the MD Anderson Dysphagia Inventory (MDADI) and the MD Anderson Symptom Inventory Head and Neck Module (MDASI-HN).

### Intervention

In both treatment arms, patients will be treated with a radical course of radiotherapy using a standard mild hypofractionation regimen of 5 fractions per week, over 4 to 6 weeks. For pragmatic reasons, each participating institution is allowed to use their standard of care dose and fractionation, but will have to be identical between the standard and experimental arms at each institution. Each center will provide, at time study entrance, what dose and fractionation will be used. This dose and fractionation will be the same for both arms and cannot change over the course of the study. The study is stratified by participating institution, which means that it will be stratified by dose/fractionation. In both treatment arms, a daily volumetric imaging method will be required for set-up verification, with match on the larynx.

#### Immobilization and simulation

Patients will be positioned in a comfortable and reproducible position and will be immobilized in a thermoplastic mask of the head and shoulders fixed to the treatment Table. A 3-dimensional planning computed tomography (CT) scan (maximum slice thickness of 1.5 mm) of the neck will be obtained. A planning magnetic resonance imaging (MRI), co-registered to the planning CT to help define gross tumor volume (GTV), is recommended but optional.

Clinicians should be aware of the risk of swallowing during CT simulation. Patients should be instructed not to swallow during the planning CT and MRI, as well as during their radiation treatment. Co-registration of the planning CT with a complementary planning volumetric imaging (such as MRI or contrast-enhanced CT) should be performed in order to verify the position of the larynx. If only one simulation imaging is obtained at time of planning, the position of the larynx should be verified on the first fraction of radiotherapy using a volumetric imaging method allowing 3D image reconstruction (Ex: Cone beam CT, CT on rail, MR-Linac).

#### Volumes

The GTV will be the same for both CL-RT and VC-RT and will be based physical examination and imaging (planning CT +/− MRI).

##### Standard arm (CL-RT)

**Fig. 2:**
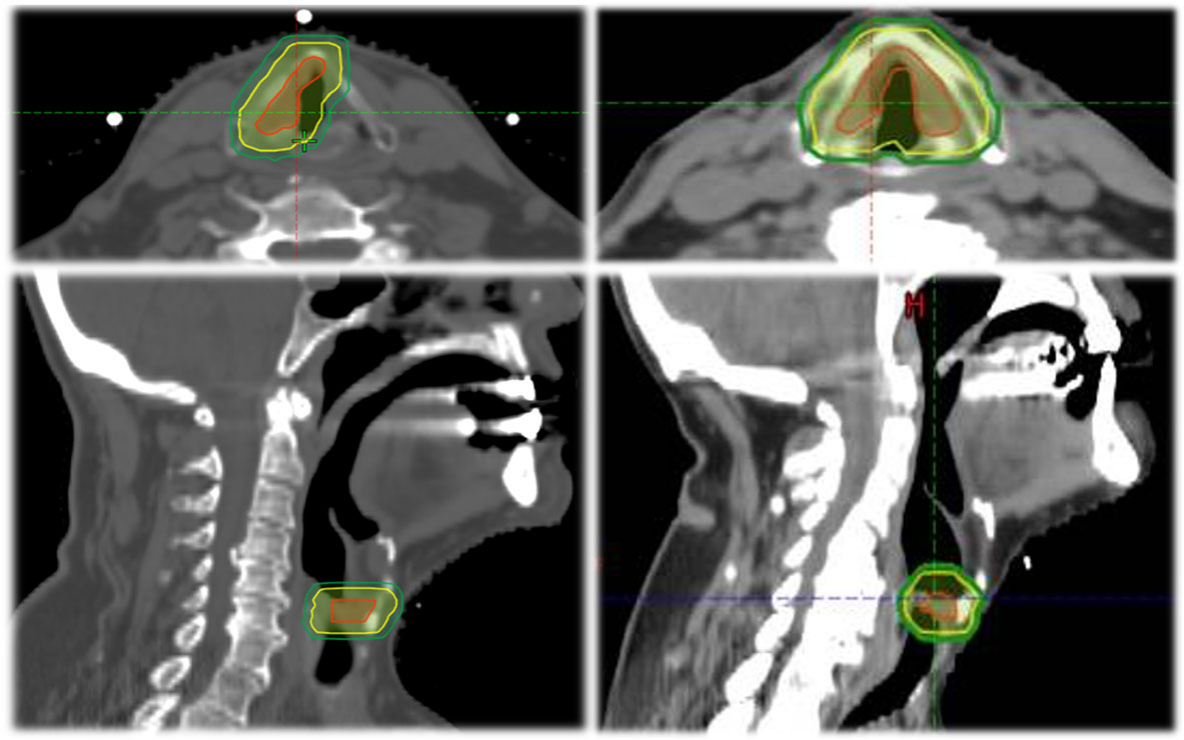
Examples of VC-RT volumes in axial and sagittal view showing for a patient with right T1a glottic cancer (LEFT) and for a patient with T1b glottis cancer (RIGHT). The entire involved vocal cord (in T1a) or both vocal cords (in T1b) form the CTV (red); the ITV formed by an isotropic margin of 6 mm around the CTV (yellow) and the PTV is formed by additional 2 mm margin (green)

The RT volume in the standard arm is based on expected fields from conventional CL-RT volume, with the planning target volume (PTV) extending from the top of thyroid cartilage lamina superiorly, to the bottom of the cricoid inferiorly. The volumes below are defined to lead to traditional volumes from conventional CL-RT (Fig. 2).

- CTV_CL-RT_ = GTV plus a manual expansion superiorly to include the cranial arytenoid cartilage, inferiorly to include 1–1.5 cm below true vocal cords, anteriorly to include the anterior commissure, posteriorly to include posterior commissure and arytenoid cartilage.
- PTV _CL-RT_ = 1 cm circumferential expansion around CTV _CL-RT_ in all directions, except posteriorly where the margin will be 0.5 cm. As the PTV of CL-RT is based on previous fields of conventional RT, after the addition of a 1 cm margin, the PTV _CL-RT_ should extend to the top of the thyroid cartilage lamina superiorly and to the bottom of the cricoid inferiorly; alternatively, the PTV _CL-RT_ should be manually expanded.

##### Experimental arm (VC-RT)

The RT volume would include the entire ipsilateral vocal cord in stage T1a disease, or both vocal cords in T1b disease. An internal target volume (ITV) consisting in an isotropic 6 mm margin will be added to account for respiratory motion. An additional margin of 2–3 mm margin will be added for setup uncertainty (Fig. 2).

- CTV_VC-RT_ = GTV plus a manual expansion to include the entire ipsilateral vocal cord in stage T1a disease, or both vocal cords in stage T1b disease. In cases where the GTV is not visualized, only a CTV (comprising the entire involved vocal cord in T1a or both vocal cords in T1b) will be contoured.
- An internal target volume (ITV) will be added and will consist in an isotropic margin of 6 mm around the CTV_VC-RT_ to account for respiratory motion.
- PTV _VC-RT_ = ITV plus a 2–3 mm margin. This is smaller than in Arm 1, because internal motion is already accounted for in the ITV.

#### Dosimetry

Use of either IMRT or protons (passive scattering or intensity modulated proton therapy) will be mandatory for all plans. Treatment plans will be normalised so that 95% of the PTV volume be covered with the prescription dose. The maximal dose to the PTV cannot exceed 110% of the prescribed dose and the dose to 99% of PTV should be above 95% of the prescribed dose (D99 > 95% of prescribed dose). Heterogeneity correction will be required for dose calculation.

#### Critical structures delineation and constraints

Critical structures delineation will be based on previously published international consensus guidelines [61]. The latter reference provides a detailed atlas of each structure and should be consulted for additional precisions. Anatomic boundaries are details in *Supplementary material *1.

##### Standard arm (CL-RT)

- Spinal cord: Not more than 0.03 cc of the planning organ at risk (PRV) exceeds 42 Gy and not more than 0.03 cc of the spinal cord receives > 40 Gy.
- Optimization of dose to other organs at risk (OAR) is not required. However, carotid-sparing is authorized.

##### Experimental arm (VC-RT)

- Spinal cord: Not more than 0.03 cc of the PRV exceeds 42 Gy and not more than 0.03 cc of the spinal cord receives > 40 Gy.
- Additional dose specification goals that should not take priority over PTV coverage:
  - ° Carotid arteries:
    - ▪ T1a
    - Ipsilateral carotid: Mean dose < 35 Gy
    - Controlateral carotid: Mean dose < 15 Gy
    - ▪ T1b
      - Mean dose to carotids < 35 Gy
  - ° Supraglottic larynx: Mean dose < 45 Gy
  - ° Contralateral vocal cord: Mean dose < 50 Gy
- Doses to other organs should be minimized and documented.

#### IGRT

A volumetric imaging method allowing 3D image reconstruction (Ex: Cone beam CT, CT on rail, MR-Linac) will be required daily for set-up verification. Additional images (e.g. confirmatory kV X-ray) may be used as supplemental verification. Larynx soft-tissue matching is required (as opposed to bony landmark [e.g. spine, base of skull] alignment).

### Study assessments

- There will be evaluation immediately post-treatment, as well as at 6, 12 and 24 months post-treatment as part of the study. Other follow-ups will be as per institution standard of care, but suggested schedule of assessments detailed in Table 1 is encouraged.
- Alternate follow-ups between the head and neck surgeon and the radiation oncologist will be mandatory.
- Physical examination will involve flexible laryngoscopy or/and videostrobe or/and mirror examination with adequate visualisation of vocal cords.
- Clinical follow-up will involve documentation of LC and survival status as well as reporting of CTCAE V4.03 toxicities.
- Patients will be asked to fill patient-reported outcome questionnaires at baseline and at each visit, as detailed in Table 1. The questionnaires will be administered in the following order: VHI-10, MDADI and MDASI-HN. The questionnaires could be filled in paper format, or online using the anonymized CASTOR EDC system. The questionnaires will not be reviewed during the study and will be analysed only at the pre-specified timepoints.
- There will be an *optional* assessment of acoustic and objective measures of voice analysis by a speech pathologist at baseline, immediately post-treatment and at 6 months post treatment. The assessments will consist in a digital recording of a standard oral reading passage and a short monologue, which would later be analyzed for specific acoustic variables and microacoustic measures of the cycle-to-cycle variation of acoustic parameters (in frequency and intensity domains). The voice quality will be graded as per then GRBAS scale (grade, roughness, breathiness, asthenia, strain).

**Table 1:** Suggested Schedule of Assessments

	Pre-RT	End of RT	2mo^a^	6mo	12 mo	24 mo	36mo^a^	48mo^a^	60mo^a^
History (including TIA or stroke) & physical exam (including laryngoscopy)	x	x	x	x	x	x	x	x	x
Side effects			x	x	x	x	x	x	x
TSH^a^	x				x	x	x	x	x
Questionnaire VHI −10	x	x	x	x	x	x	x	x	x
Questionnaire MDADI and MDASI-HN	x	x	x	x	x	x	x	x	x

*RT* Radiotherapy, *Mo* months

^a^Optional assessments

### Statistical considerations

According to previous experience, expected LC after CL-RT is estimated to be 92% [6, 8]. Assuming a non-inferiority margin of 8%, if LC in the VC-RT arm is ≥84% at 2-years, we will deem the VC-RT arm acceptable. With a sample size of 111 patients, over 82% power will detect a margin difference of 8%, at a target significance level of 0.05 using a one-sided, exact binomial test (NCSS-PASS 2005). The purpose of the randomization will be to generate currently unavailable data on secondary outcomes of voice impairment, dysphagia and quality of life in the CL-RT arm. Considering a 5% attrition rate, 155 patients will be randomized in a 1:3 ratio to CL-RT (39 patients) and VC-RT (116 patients) arms. Patients will be stratified by stage (T1a/T1b) and by institution.

An interim analysis will be conducted when 55 patients have a minimal follow-up of 6 months. We will monitor the efficacy endpoint using the Bayesian optimal phase 2 (BOP2) design [62]. Specifically, let *n* denote the interim sample size and *N* denote the maximum sample size. Let *p*_*eff*_ denote the probability of efficacy (response rate) and define the null hypothesis *H*_0_ : *p*_*eff*_ < 0.84, representing that the treatment is inefficacious. We will stop enrolling patients and claim that the treatment is not promising if

$$ \mathit{\Pr}\left({p}_{eff}\ge 0.84\mid data\right)<\lambda {\left(\frac{n}{N}\right)}^{\alpha }, $$

where *λ* =0.95 and *α* =1 are design parameters optimized to minimize the chance of incorrectly claiming that an efficacious treatment is not promising (i.e., type II error) under the alternative hypothesis *H*_1_ : *p*_*eff*_ = 0.92, while controlling the type I error rate at 0.05 (i.e., the chance of incorrectly claiming that an inefficacious treatment is promising is no more than 5%). Assuming a Beta (0.84,0.16) prior distribution for *p*_*eff*_, if more than 10 patients among the 55 patients develop recurrences, the trial will be stopped in the interim analysis. Otherwise, the trial will be continued.

### Analytic plan

A Bayesian regression model [63, 64] will be used to compare the LC between the VC-RT and CL-RT arms at 2 years. The LC and OS time will be defined from end of RT date. The following recurrences will be considered LC: in field, field margin and within larynx. Local recurrences will be biopsy-proven when possible. Competing events will include local recurrence, regional/distant recurrence and death prior to recurrence. LC will be estimated using both competing risk and Kaplan -Meier (KM) analysis. The analyses will be conducted using SAS and R software. A Cox multivariable regression analysis will be used to determine factors predictive of LC, PFS and OS. Chi-squared test or Fisher’s exact test will be used to compare the difference in toxicity between arms. For the secondary endpoints of patient-reported outcomes (VHI-10, MDADI and MDASI-HN), trajectory trends of scores between/among arms over time will be explored using a generalized linear mixed model with a random effect of time. Appropriate adjustments for covariates will be considered.

There will be two planned analysis: 1) an interim analysis and, 2) a final analysis. The interim analysis will be done after 55 patients on the experimental arm have a minimum follow-up of 6 months. A short follow-up time of 6 months was chosen for the interim analysis for safety (i.e. for early detection of local recurrence trends). The trial may be interrupted early if VC-RT is deemed not promising on interim analysis. The final analysis will performed 2 years after accrual is completed.

### Data safety and monitoring board

A data safety and monitoring board (DSMB) from the Centre Hospitalier de l’Université de Montréal will be responsible for assessing the safety data. The trial would be interrupted early if higher than expected rates of local recurrences are observed. The DSMB will review interim/cumulative evidence of study related safety, consider factors external to the study when relevant information becomes available and provide the sponsor a recommendation as to whether the study should: continue without change, be modified, suspended or terminated. The DSMB will assess the safety data twice a year. An interim analysis will be planned after the first 55 patients in the experimental arm have a 6-month follow-up. Using a Bayesian probabilistic model, the trial would be interrupted early if higher than expected rates of local recurrences are observed. As this trial involves only a reduction of RT treatment volumes, we do not expect any increase in toxicities in patients treated on the experimental arm.

### Subject discontinuation / withdrawal

Patients may withdraw from the study prior to the completion of study related procedures for the following reasons:

- Patient withdraws consent for participation. Subjects may voluntarily discontinue participation in the study at any time.
- It is deemed in the patient’s best interest as determined by the attending/principal investigator.

### Consent and confidentiality

All enrolled patients will be required to sign informed consent before study entry. The principal investigator, the co-investigators and the research nurses will gather and record all collected information in a research record. All information collected will remain strictly confidential to the extent permitted by law. In all research records, subjects will be identified by enrolment number.

### Protocol amendments and trial publication

The protocol, the informed consent form, and any other written information to be given to subjects will be reviewed and approved by a properly constituted Institutional Review Board (IRB)/Research Ethics Board (REB), operating in accordance with federal laws and regulations. Any institution opening this study will obtain REB IRB/REB approval prior to local initiation. Any amendment to the study will be submitted for review by the IRB/REB before any changes are implemented unless required to eliminate immediate hazard to the study participants.

The results of this trial will be reported in scientific publications as well as scientific conferences. Patients will not be indicated by name. The protocol, results from interim analysis and final results will be published in peer-reviewed journals.

## Discussion

Swallowing motion over the course of early glottic radiotherapy has been reported to be rare and unlikely to impact on dose delivery [50, 53, 55]. However, respiratory motion, laryngeal shift in relation to the vertebral structures over the course of RT, as well as the occasional occurrence of swallowing motion at the time of simulation could introduce substantial treatment delivery inaccuracies [50, 57]. The safety of VC-RT may therefore be highly dependent on adequate precautions including the addition of an ITV margin accounting for breathing motion, use of volumetric IGRT with daily match on the larynx as well as verification of larynx resting position at time of planning CT scan acquisition. Following these principles, the group from Erasmus MC Cancer Institute reported excellent outcomes from VC-RT in a retrospective cohort 30 patients with T1a glottic cancer, with no local failures at 2 years [35]. More recently, Sher et al. reported outcomes from a phase I dose-escalation trial (50 Gy in 15 escalated to 42.5 Gy in 5 fractions) of VC-RT for Tis-T2N0 of the glottis, using an ITV derived from a 4D-CT [65]. While the study reported acceptable early tolerability of the ultra-hypofractionated regimen with 7% rate of dose limiting grade 3–4 toxicity, as high as 17% of patients (5 of 29) developed a local recurrence. A closer look at these 5 recurrences reveals that 3 were among 7 patients with T2 tumors (leading to a 43% local recurrence rate in T2N0), 1 occurred in a patient with a T1b lesion that was inaccurately delineated and resulted in a marginal miss, and 1 was in a previously understaged tumor which, in retrospect, was a T4 lesion. While these results are somewhat reassuring in regard to the safety of VC-RT for T1N0 glottic larynx, these recurrences certainly highlight the importance of careful staging and accurate clinical assessment of tumor extension when considering VC-RT, and certainly question the safety of this approach for T2 tumors. Total laryngectomy frequently constituting the salvage treatment option in the context of local recurrence after irradiation, the consequences of treatment failures can have severe impacts on patients’ quality of life and survival. Taken together, these considerations justify the current equipoise as to whether VC-RT can lead to similar efficacy as CL-RT in early glottic cancer.

The current phase II multicenter Bayesian trial will accrue 155 patients with T1N0 glottic cancer, randomized to VC-RT versus CL-RT. The study is limited by the fact that it does not constitute a true randomized non-inferiority trial, but the feasibility of such a design is rendered impossible by the unrealistic necessary sample size of > 2000 patients. Therefore, this trial would likely provide the best possible comparison between the 2 treatment arms. Local control at 2 years has been selected as the primary endpoint as the majority of local recurrences occur within the first 2 years after radiotherapy [66, 67], however, LC will continue to be monitored in the context of this trial up to 5 years after radiotherapy. LC in the literature for T1N0 of the glottic larynx varies between 85 and 95% [6–8, 24, 25].; this variation is at least partly dependent on the distribution of T1a versus T1b in the cohorts. With a target LC of 92% and a margin of 8%, any LC below 84% - therefore below the expected range- would be deemed unacceptable. In the context of this pragmatic trial, each center will use their own standard of care dose and fractionation regimen; as a general principle, radiotherapy will consist in mild hypofractionation regimen of 5 fractions per week, over 4 to 6 weeks. Institutions will not be allowed to change their dose/fractionation once accrual has started. As the study is stratified by participating institution, there will be a balanced number of patients with each dose/fractionation in each arm. In addition, as intensity modulated proton beam therapy is the standard of care for early glottic larynx cancer in some centers, the trial allows the use of proton beam therapy, as long as the same treatment technique is used in each arm.

The delivery of VC-RT will be guided by cautious principles to maximize the safety of treatment delivery which will include: careful definition of tumor extension in collaboration with a head and neck surgeon, use of planning MRI where possible for optimal tumor staging and delineation, use of an ITV for breathing motion, as well as mandatory daily volumetric IGRT. The primary endpoint of the trial will be LC, with secondary endpoints of quality of life (including voice, dysphagia and head and neck symptom burden), toxicity as well as objective measures of voice. If positive, this would result in a paradigm shift in the approach to radiation for treatment early glottic cancers in the institutions where CL-RT remains the standard. On the contrary, if VC-RT demonstrates inferior LC compared to CL-RT, the results of this trial would strengthen the necessity to maintain conservative margins until the possible loopholes in the safety of VC-RT are better understood.

## Supplementary Information

**Additional file 1.**

## Data Availability

Not applicable.

## Abbrevations

4D-CT: Four-dimensional computed tomography
BOP-2: Bayesian optimal phase 2
GRBAS: Grade, roughness, breathiness, asthenia, strain
CL-RT: Complete larynx radiotherapy
CT: Computed tomography
CTCAE: Common toxicity criteria for adverse events
CTV: Clinical target volume
DSMB: Data safety and monitoring board
ECOG: Eastern Cooperative Oncology Group
GTV: Gross tumor volume
HNC: Head and neck cancer
IGRT: Image-guided radiotherapy
IMRT: Intensity modulated radiotherapy
IRB: Institutional Review Board
ITV: Internal target volume
KM: Kaplan meier
LC: Local control
MDADI: MD Anderson dysphagia inventory
MDASI-HN: MD Anderson Symptom inventory- Head and neck module
MRI: Magnetic resonance imaging
OS: Overall survival
PTV: Planning target volume
PRV: Planning organ at risk volume
REB: Research Ethics Boar
RT: Radiotherapy
VC-RT: Vocal cord radiotherapy
VHI-10: Voice handicap index-10
