# Supplementary material for: Vocal-cord Only vs. Complete Laryngeal radiation (VOCAL): a randomized multicentric Bayesian phase II trial
Source: BMC Cancer. 2021 Apr 22;21:446. doi: 10.1186/s12885-021-08195-8 (PMC8061218; doi:10.1186/s12885-021-08195-8)
Supplement: Supplementary file 1 — Additional file 1. [file 12885_2021_8195_MOESM1_ESM.docx]

***SUPPLEMENTARY MATERIAL 1***

***Organs at risk delineation guidelines***

- ***Spinal cord:*** The spinal cord is delineated as the true spinal cord, not the spinal canal. The cranial border is defined at the tip of the dens of C2 (the lower border of the brainstem), and the caudal border at the upper edge of T3. Spinal cord contours will be extended by at least 5 cm caudal to the PTV. A 0.5 cm margin will be added around the spinal cord to create a *Planning Organ at Risk Volume* (PRV).
- ***Supraglottic larynx*:** The cranial border will be at tip of epiglottis and caudal border as the cranial edge of arytenoid cartilages. The pharyngeal lumen will be excluded.
- ***Controlateral arytenoid****:* Arytenoids are recognized as are paired, pyramidal cartilages that are attached posteriorly to the vocal cords. The caudal edge is at the articulation with the cricoid cartilage. Only the contralateral arytenoid will require to be contoured.
- ***Pharyngeal constrictor muscles:*** The cranial border is defined as the caudal tip of pterygoid plates and the caudal border as the lower edge of the cricoid cartilage. A 3 mm antero-posterior thickness should be used for contouring of the structure.
- ***Contralateral vocal cord:*** The entire contralateral vocal cord, extending anteriorly from the thyroid cartilage (to include anterior commissure) to the arytenoid cartilage (to include posterior commissure) should be contoured. Contour of the contralateral vocal cord should not overall with the contralateral arytenoid.
- ***Right and left common carotid arteries:***  These will be defined as 2 separate structures. The carotid artery will include the common and internal carotid artery. The right common carotid originates at the brachiocephalic trunk and the left carotid artery at the aortic arch. Cranially, the common carotid artery will end at the bifurcation into the external and internal carotid arteries (around the level of C4).
- ***Thyroid gland:*** The thyroid gland is located below the thyroid cartilage. The two connected lobes and the isthmus should be included in the structure.
- ***Submandibular glands:*** The cranial border will be defined as the medial pterygoid and mylohyoid muscles, the caudal border will be at the end of visible submandibular gland, the anterior border will be at the surface mylohyoid and hyoglossus muscles, the posterior border will be formed by the parapharyngeal space and sternocleidomastoid muscle, the lateral border will be the medial surface of medial pterygoid muscle and medial surface of mandibular bone and platysma most inferiorly; the medial border will be the lateral surface of the mylohyoid, hyoglossus muscles and anterior belly of the digastric muscle.
- ***Cervical esophagus:*** The cervical esophagus will be defined superiorly 1 cm caudal to the lower edge of the cricoid cartilage, and inferiorly at the caudal edge of C7.
